# Supplementary material for: Modeling soybean growth: A mixed model approach
Source: PLoS Comput Biol. 2024 Jul 11;20(7):e1011258. doi: 10.1371/journal.pcbi.1011258 (PMC11265664; doi:10.1371/journal.pcbi.1011258)
Supplement: S1 File — The section entitled “Algorithmic details” describes the SAEM algorithm key distributions and the algorithm steps for both parameter estimation (Algorithm A) and genetic effects prediction (Algorithm B) for anyone wishing to implement the methodology described in the present article. Section “Convergence graphs” provides convergence graphs showing that the SAEM algorithm converges well in practice in the analysis of real soybean growth data presented in section 3.2 of the article: Fig A, Fig B, Fig C, Fig D. (PDF) [file pcbi.1011258.s001.pdf]

Supporting information for the article "Modeling soybean growth: A mixed model approach"

Maud Delattre [1*], Yusuke Toda[2], Jessica Tressou[2,3], Hiroyoshi Iwata[2].

1 Université Paris-Saclay, INRAE, MaIAGE, Jouy-en-Josas, France

2 Graduate School of Agricultural and Life Sciences,The University of Tokyo, Tokyo, Japan

3 Paris-Saclay University-AgroParisTech-INRAE, UMR MIA-Paris-Saclay, Palaiseau, France

* maud.delattre@inrae.fr

1 Algorithmic details.

The objective of this appendix is to provide the main technical elements for implementing the SAEM algorithm in model (1)-(2). First, we introduce the compact matrix notations:

- $\varphi = \begin{pmatrix} \varphi_1 \\ \vdots \\ \varphi_{N_p} \end{pmatrix}$: the $m.N_p$ vector obtained by concatenation of the φ_i's,

- $X = \begin{pmatrix} X_1 \\ \vdots \\ X_{N_p} \end{pmatrix}$ (resp. $Z = \begin{pmatrix} Z_1 \\ \vdots \\ Z_{N_p} \end{pmatrix}$): the $m.N_p \times p$ (resp. $m.N_p \times q.N_v$) matrix obtained by vertical concatenation of the X_i's (resp. Z_i's),

1. **Key distributions in model (1)-(2)**

   (a) **Complete data log-likelihood.**

   Using that in model (1)-(2), $y_{ij}|\varphi_i, u \sim \mathcal{N}(g(t_{ij}, \varphi_i), \sigma^2)$, $\varphi|u \sim \mathcal{N}(X\beta + Zu, P_{N_p})$ and

$u \sim \mathcal{N}(\mathbf{0}, K \otimes G)$, we get

$$\begin{aligned}\log p(y,\varphi,u;\theta) &= \log p(y \mid \varphi,u;\theta) + \log p(\varphi \mid u;\theta) + \log p(u;\theta) \\ &= A - \frac{N_{tot}}{2}\log\sigma^2 - \frac{1}{2\sigma^2}\sum_{i=1}^{N_p}\sum_{j=1}^{n_i}(y_{ij} - g(t_{ij},\varphi_i))^2 - \frac{N_p}{2}\log(|P|) \\ &- \frac{1}{2}(\varphi - Zu - X\beta)'(I_{N_p}\otimes P^{-1})(\varphi - Zu - X\beta) - \frac{1}{2}\log(|K\otimes G|) \\ &\qquad - \frac{1}{2}u'(K\otimes G)^{-1}u, \end{aligned} \quad (1)$$

where A is a constant term which does not depend on θ, I_{N_p} is the identity matrix of size N_p, n_i is the number of observations for plant $i \in \{1,\dots,N_p\}$ and $N_{tot} = \sum_{i=1}^{N_p} n_i$ is the total number of observations available in the sample (*i.e.* all observations of all plants).

(b) **Conditional distribution of the genetic effects u given φ.**

Using the Bayes formula where $p(u|\varphi;\theta) = p(\varphi|u;\theta)p(u;\theta)/p(\varphi;\theta)$ and that in model (1)-(2), $\varphi|u \sim \mathcal{N}(X\beta + Zu, P_{N_p})$, $u \sim \mathcal{N}(\mathbf{0}, K\otimes G)$ and
$\varphi \sim \mathcal{N}(X\beta, Z(K\otimes G)Z^\top + I_{N_p}\otimes P)$, by elementary calculations, we get that
$u|\varphi \sim \mathcal{N}(m_u(\theta), \Sigma_u(\theta))$, where

$$\begin{aligned}\Sigma_u(\theta)^{-1} &= Z^\top(I_{N_p}\otimes P^{-1})Z + (K\otimes G)^{-1} \\ m_u(\theta) &= \Sigma_u(\theta)Z(I_{N_p}\otimes P^{-1})(\varphi - X\beta) \end{aligned} \quad (2)$$

2. **SAEM algorithm**

Algorithm 1 describes the algorithm used to estimate parameters. K is the total number of iterations, which is tuned by the user such that the algorithm converges at the end of K iterations. The stochastic approximation step uses model (1)-(2) belonging to the curved exponential family, and thus consists of the computation of the stochastic approximations of the sufficient statistics.

Remarks:

- To better manage the acceptance proportions at the simulation step of the algorithm, it is possible to perform several (but a finite number of) iterations of Metropolis-Hastings rather than one, and define $\varphi_i^{[k]}$ as the last accepted value after these iterations.

- Choosing $q_{i,\theta}(\varphi)$ as $p(\varphi|u;\theta)$ allows to simplify the expression of the acceptance ratio $\alpha_{i,k}$.
- In some situations, in particular when the number of observed plants per variety is small, the convergence of the algorithm can be improved by using M Monte-Carlo Markov chains instead of only one as described in Algorithm 1. This amounts to simulating M values $\tilde{\varphi}_i^{[k,m]}$, $m \in 1, \ldots, M$, at each iteration and replacing the sufficient statistics by their means over the M simulated values.

Algorithm 2 presents algorithm for genetic effects prediction. This should be used with $\theta = \hat{\theta}^{[K]}$ obtained at the convergence of the first algorithm. In general, Algorithm 2 is written for any value of θ. It also exploits the fact that the model (1)-(2) belongs to the curved exponential family.

Algorithm A SAEM algorithm for parameter estimation

Input: $(\gamma_k)_{k\geqslant 0}$

Initialization: $\theta^{[0]}$, $u^{[0]}$, $\varphi^{[0]}$

for $k \in \{1,\ldots,K\}$ **do**

1. S-step

for $i \in \{1,\ldots,N_p\}$ **do**

Draw $\tilde{\varphi}_i^{[k]}$ from a proposal kernel $q_{i,\theta^{[k-1]}}(\varphi)$ and set $\varphi_i^{[k]} = \tilde{\varphi}_i^{[k]}$ with probability

$$\begin{aligned}\alpha_{i,k} &= \min\left(1, \frac{p(\tilde{\varphi}_i^{[k]}|u^{[k-1]}, y_i; \theta^{[k-1]})q_{i,\theta^{[k-1]}}(\varphi_i^{[k-1]})}{p(\varphi_i^{[k-1]}|u^{[k-1]}, y_i; \theta^{[k-1]})q_{i,\theta^{[k-1]}}(\tilde{\varphi}_i^{[k]})}\right)\\ &= \min\left(1, \frac{p(y_i|\tilde{\varphi}_i^{[k]}; \theta^{[k-1]})p(\tilde{\varphi}_i^{[k]}|u^{[k-1]}; \theta^{[k-1]})q_{i,\theta^{[k-1]}}(\varphi_i^{[k-1]})}{p(y_i|\varphi_i^{[k-1]}; \theta^{[k-1]})p(\varphi_i^{[k-1]}|u^{[k-1]}; \theta^{[k-1]})q_{i,\theta^{[k-1]}}(\tilde{\varphi}_i^{[k]})}\right)\end{aligned}$$

and $\varphi_i^{[k]} = \varphi_i^{[k-1]}$ with probability $1-\alpha_{i,k}$.

end for

Draw $u^{[k]}$ under a Gaussian distribution with mean $m_u(\theta^{[k-1]})$ and covariance matrix $\Sigma_u(\theta^{[k-1]})$ whose formula are given in (2).

2. SA-step

Compute stochastic approximations of the sufficient statistics

$$\begin{aligned}s_1^{[k]} &= (1-\gamma_k)s_1^{[k-1]} + \gamma_k(\varphi^{[k]} - Zu^{[k]})\\ s_2^{[k]} &= (1-\gamma_k)s_2^{[k-1]} + \gamma_k(u^{[k]})^\top K^{-1}u^{[k]}\\ s_3^{[k]} &= (1-\gamma_k)s_3^{[k-1]} + \gamma_k(\varphi^{[k]} - Zu^{[k]})^\top(\varphi^{[k]} - Zu^{[k]})\\ s_4^{[k]} &= (1-\gamma_k)s_4^{[k-1]} + \gamma_k\sum_{i=1}^{N_p}\sum_{j=1}^{n_i}(y_{ij} - g(t_{ij}, \varphi_i^{[k]}))^2\end{aligned}$$

3. M-step

Update the parameter estimates according to

$$\begin{aligned}\hat{\beta}^{[k]} &= (X^\top(I_{N_p}\otimes(P^{[k-1]})^{-1})X)^{-1}X^\top(I_{N_p}\otimes(P^{[k-1]})^{-1})s_1^{[k]}\\ \hat{P}^{[k]} &= \frac{1}{N_p}\left(s_3^{[k]} - 2(X\hat{\beta}^{[k]})^\top s_1^{[k]} + (X\hat{\beta}^{[k]})^\top X\hat{\beta}^{[k]}\right)\\ \hat{G}^{[k]} &= \frac{s_2^{[k]}}{N_v}\\ \widehat{\sigma^2}^{[k]} &= \frac{s_4^{[k]}}{N_{tot}}\end{aligned}$$

end for

Output: $\hat{\theta}^{[K]} = (\hat{\beta}^{[K]}, \hat{P}^{[K]}, \hat{G}^{[K]}, \widehat{\sigma^2}^{[K]})$.

Algorithm B SAEM algorithm for genetic effects prediction

Input: $(\gamma_k)_{k\geqslant 0}$, $\theta = (\beta, P, G, \sigma^2)$

Initialization: $u^{[0]}$, $\varphi^{[0]}$

for $k \in \{1, \ldots, K\}$ **do**

1. S-step

for $i \in \{1, \ldots, N_p\}$ **do**

Draw $\tilde{\varphi}_i^{[k]}$ from a proposal kernel $q_{i,\theta}(\varphi)$ and set $\varphi_i^{[k]} = \tilde{\varphi}_i^{[k]}$ with probability

$$\begin{aligned}\alpha_{i,k} &= \min\left(1, \frac{p(\tilde{\varphi}_i^{[k]}|u^{[k-1]}, y_i; \theta) q_{i,\theta}(\varphi_i^{[k-1]})}{p(\varphi_i^{[k-1]}|u^{[k-1]}, y_i; \theta) q_{i,\theta}(\tilde{\varphi}_i^{[k]})}\right) \\ &= \min\left(1, \frac{p(y_i|\tilde{\varphi}_i^{[k]}; \theta) p(\tilde{\varphi}_i^{[k]}|u^{[k-1]}; \theta) q_{i,\theta}(\varphi_i^{[k-1]})}{p(y_i|\varphi_i^{[k-1]}; \theta) p(\varphi_i^{[k-1]}|u^{[k-1]}; \theta) q_{i,\theta}(\tilde{\varphi}_i^{[k]})}\right)\end{aligned}$$

and $\varphi_i^{[k]} = \varphi_i^{[k-1]}$ with probability $1 - \alpha_{i,k}$.

end for

2. SA-step

Compute stochastic approximations of the sufficient statistics

$$s^{[k]} = (1 - \gamma_k) s_1^{[k-1]} + \gamma_k \varphi^{[k]}$$

3. M-step

Update the genetic values according to

$$\hat{u}^{[k]} = \left(Z^\top (I_{N_p} \otimes P^{-1}) Z + (K \otimes G)^{-1}\right)^{-1} Z^\top (I_{N_p} \otimes P^{-1})(s^{[k]} - X\beta)$$

end for

Output: $\hat{u}^{[K]}$.

2 Convergence graphs.

[Fig 1 about here.]

[Fig 2 about here.]

[Fig 3 about here.]

[Fig 4 about here.]

List of Figures

A Real data analysis: Parameter estimation. Evolution of β's and σ^2's estimations over iterations of the SAEM algorithm. 8
B Real data analysis: Parameter estimation. Evolution of P's estimation over iterations of the SAEM algorithm. 9
C Real data analysis: Parameter estimation. Evolution of the estimation of the diagonal terms of G over iterations of the SAEM algorithm. 10
D Real data analysis: Parameter estimation. Evolution of the estimation of the extra-diagonal terms of G over iterations of the SAEM algorithm. 11

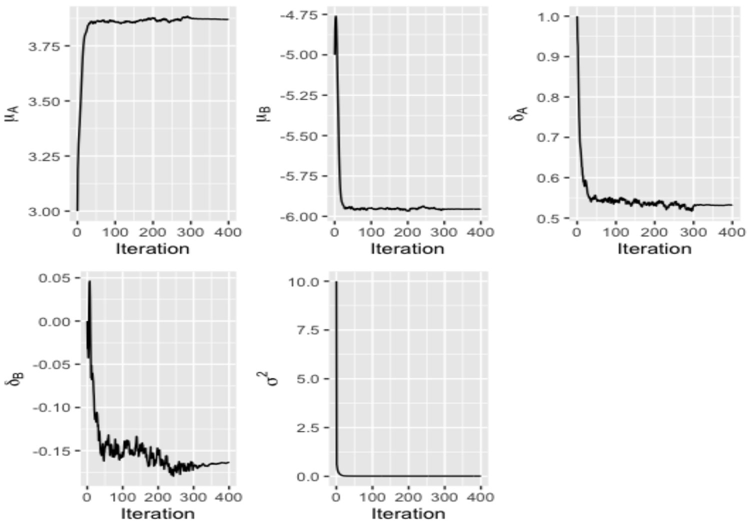

Fig A. Real data analysis: Parameter estimation. Evolution of β's and σ^2's estimations over iterations of the SAEM algorithm.

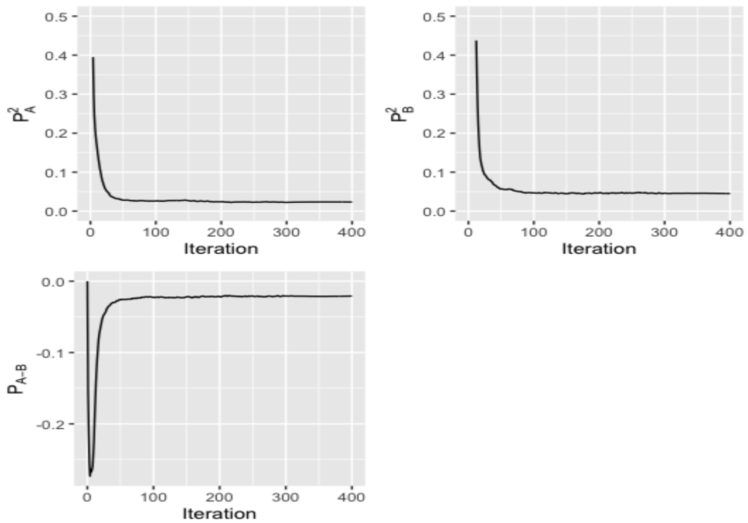

Fig B. Real data analysis: Parameter estimation. Evolution of P's estimation over iterations of the SAEM algorithm.

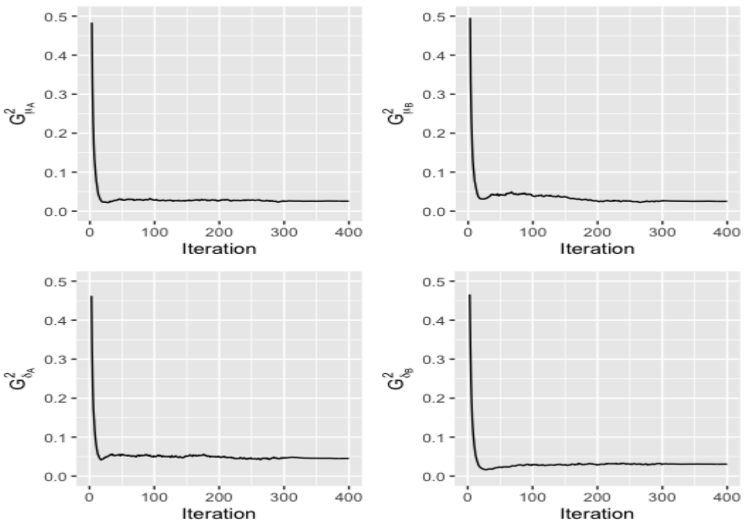

Fig C. Real data analysis: Parameter estimation. Evolution of the estimation of the diagonal terms of G over iterations of the SAEM algorithm.

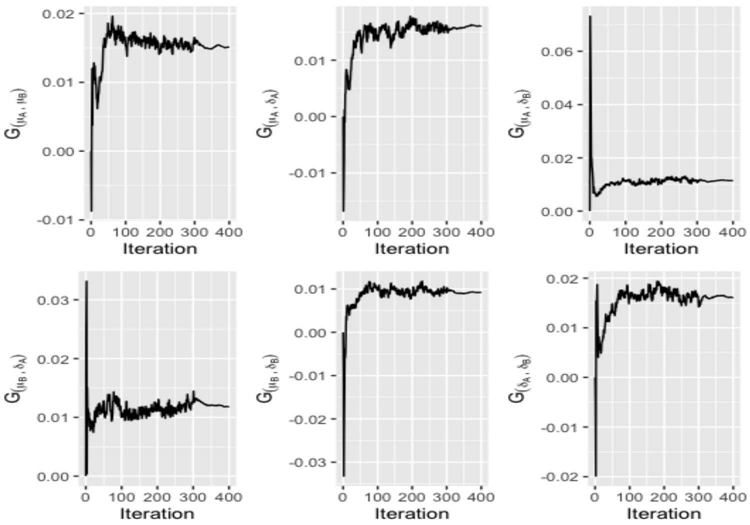

Fig D. Real data analysis: Parameter estimation. Evolution of the estimation of the extra-diagonal terms of G over iterations of the SAEM algorithm.
